# Recall and awareness of gambling advertising and sponsorship in sport in the UK: a study of young people and adults

**DOI:** 10.1186/s12954-019-0291-9

**Published:** 2019-04-02

**Authors:** Natalie Djohari, Gavin Weston, Rebecca Cassidy, Martyn Wemyss, Samantha Thomas

**Affiliations:** 1 Department of Anthropology, Goldsmiths, University of London, New Cross, London, SE14 6NW UK; 2 Centre for Population Health Research, School of Health and Social Development, Faculty of Health, Deakin University, Geelong, Australia

**Keywords:** Gambling, Young people, Children, Advertising, Sport, Betting

## Abstract

**Background:**

The impact of gambling advertisements shown during sporting events on young people is an important public health issue. While extensive research has taken place in Australia, there is still only a limited understanding of this issue in the United Kingdom (UK).

**Method:**

A mixed methods study was conducted with 71 family groups comprised of 99 young people (8–16 years) and 71 adults recruited at six sites across South London, England (May–July 2018). Interviewer-assisted surveys investigated recall and awareness of sports betting brands using interviews and a magnet placement board activity developed in Australia. Quantitative data were analysed using descriptive statistics, with qualitative data interpreted using thematic analysis techniques.

**Results:**

Just under half of young people (*n* = 46, 46%) and more than two thirds of adults (*n* = 49, 71%) were able, unprompted, to name at least one gambling brand. Boys had a significantly higher recall of brands than girls, as did young people who watched a lot of football on television. Almost two thirds of young people (*n* = 63, 63%) correctly placed one or more shirt sponsor magnets next to the corresponding football team, and 30% (*n* = 30) correctly placed three or more sponsors magnets next to the corresponding football team. Just under two thirds of adults (*n* = 44, 62%) correctly placed one or more shirt sponsors magnets next to the corresponding football team. Young people recalled seeing gambling advertising on television (*n* = 78), technology/screens (*n* = 49), and in association with sports teams (*n* = 43). Adults recalled seeing advertising on television (*n* = 56), on technology/screens (*n* = 37), in sports stadiums (*n* = 34), and in betting venues (*n* = 34). Over three quarters of young people (*n* = 74 out of 95 responses, 78%) and 86% of adults (*n* = 59 out of 69 responses) thought that betting had become a normal part of sport.

**Conclusion:**

In order to reduce the exposure of young people to gambling advertising, policymakers in the UK should consider comprehensive approaches, similar to those applied in tobacco control, which cover all forms of advertising, including promotion and sponsorship.

## Background

### Gambling advertising in the UK

The impact of gambling advertisements shown during sporting events is increasingly recognised by the international research community as an important public health issue for young people [1–3]. Much of the research investigating the impact of gambling advertising on young people has been conducted in Canada and Australia, while there is still only a limited understanding of this issue in the United Kingdom (UK).

Gambling advertising in the UK has increased rapidly since the Gambling Act 2005 came into force in 2007. The number of ‘spots’ advertising gambling on television increased from 152,000 in 2006 to 1.39 million in 2012 [4]. The amount gambling companies spend on advertising has also increased. In 2015, gambling companies spent £118.5m on television advertising, an increase of 46% since 2012 [5]. In 2018, bookmaker Paddy Power estimated that advertising spend in the sports betting market was rising at a rate of 19% per annum [6].

Oversight of gambling advertising in the UK is the responsibility of the Department for Digital, Culture, Media and Sport (DDCMS), Ofcom, and the UK Gambling Commission. Gambling operators must also comply with advertising codes of practice (BCAP—The UK Code of Broadcast Advertising, and CAP—the UK Code of Non-broadcast Advertising and Direct & Promotional Marketing) which are administered by the Advertising Standards Authority (ASA). Operators have also created the Industry Code for Socially Responsible Advertising ([7], pg. 25). The ASA codes focus on content restrictions: in particular, that gambling advertisements must not ‘be likely to be of particular appeal to children or young persons, especially by reflecting or being associated with youth culture’ [8].

While most gambling advertising (with the exception of bingo) is prohibited before the 9 pm watershed in the UK, sports betting may be promoted at any time of the day during live sport [9]. Furthermore, and in common with other jurisdictions such as Australia, advertising restrictions do not apply to sponsorship promotions, such as shirt logos and signage [10]. A recent study of televised football matches on Sky Sports and the BBC found that a majority of advertisements (29 out of 30 gambling brands and 57 unique brands overall) were for online gambling, with the majority of these advertisements appearing on dynamic billboards on the sides of football pitches [11].

With increasing public concern about the impact of gambling advertising on young people, there have been some shifts in sponsorship relationships between sporting codes and gambling companies. The Football Association (the governing body of association football in England) ended its sponsorship deal with Ladbrokes in 2017, and the English Premier League (EPL) has not sought sponsorship since the 2016–17 season. However, in the 2018–19 season, nine out of the 20 EPL teams and 17 out of 24 Championship sides had gambling firms as shirt sponsors [12].

### The impact of gambling advertising, promotions, and sponsorship on young people

Research has demonstrated that sponsorship promotions have a powerful impact on young people’s awareness, attitudes, and consumption intentions towards unhealthy commodity products such as tobacco, alcohol, and gambling [2, 3, 13–17]. Recent studies in New Zealand and Australia have explored the prevalence of gambling sponsors in sport [18], young people’s recall of gambling brands [3, 19], the influence and impact of gambling advertisements on young people’s gambling attitudes and decision making [20], and young people’s recall of gambling advertising at different times of day and on different media channels [21]. These studies have demonstrated that young people, and particularly those who are fans of sport, have a high recall of gambling brand names, see gambling advertising on multiple media channels (including on social media), may have a reduced perception of the risks associated with gambling because of the messages within advertising campaigns, and perceive that gambling is a normal or common part of sport [2, 19, 21]. The studies also suggest that young people and adults want increased action on advertising to protect young people from exposure to advertising for these products [21, 22].

Studies have also investigated young people’s recall of gambling sponsorship, relative to their awareness of junk food and alcohol sponsors. In an Australian study of 85 young people aged 5–12 years, three quarters were able to identify at least one correct shirt sponsor associated with food, alcohol, or gambling companies [23]. In Australia, there have been initiatives to remove gambling sponsorship from professional sport, with Victorian teams in the Australian Football League among those who have signed a charter with the Victorian Responsible Gambling Foundation which commits them to reject sponsorship from gambling companies [24]. While concerns about the potential impact of gambling advertising on young people have been raised by the media in the UK [5, 25], less research has taken place in this jurisdiction.

The following study aimed to contribute to the international literature on the impact of gambling advertising, by exploring how young people and adults recall gambling brands that are advertised and promoted within sport in the UK. The research was guided by five research questions:

1. To what extent do young people and adults recall sports betting brands?
2. Are young people and adults able to implicitly recall sponsorship relationships between gambling brands and teams playing in the EPL?
3. To what extent do young people and adults perceive gambling as a normal part of sport?
4. Where, if anywhere, do young people and adults see gambling advertising?
5. What changes, if any, do young people and adults want to see to the current regulation of gambling advertising?

## Methods

### Approach

Adapting the methods created in Australia [3, 17, 23], this study used a mixed methods approach to explore young people’s and adult’s recall of sports betting brands, their implicit recall of sponsorship relationships between gambling brands and teams playing in the EPL, where young people and adults see gambling advertising, the extent to which young people and adults perceive gambling as a normal part of sport, and what changes, if any, young people and adults want to see to the current regulation of gambling advertising.

In order to assess the implicit associations of adults and young people between professional sporting teams and their sponsors, the study drew upon the research of Pettigrew et al. [17], Bestman et al. [23], and Thomas et al. [3], which used projective techniques. The other questions were answered using more traditional survey questions. Higher risk ethical approval was received from the Goldsmiths University Human Research Ethics Committee (Goldsmiths 1365), given that young people were involved and sports betting is illegal for children under 18 in the UK.

### Sampling and recruitment

The study recruited a convenience sample of family groups comprised of at least one 8- to 16-year-old and at least one parent or carer. This age range has been used in similar studies and represents the time at which young people become aware of gambling marketing and are able to understand the persuasive intent of marketing [3]. Young people were recruited between May and July 2018 at six sites across South London. This time period was chosen in order to coincide with events which take place outdoors and attract young people and accompanying adults from a range of backgrounds, including those with varying levels of engagement and interest in football. Events included a young people’s football tournament run by a junior football club, three community festivals, a family car boot sale, and a primary school family camping event. While in Australian studies, major sporting events have been used as primary research locations for data collection, we were unable to replicate this as all of the London-based EPL, Championship, and League One teams who we approached either declined or did not respond to our requests for access to stadiums to conduct the study.

Up to 15 undergraduate and postgraduate researchers (trained and supported by GW and ND) attended each event. A senior colleague (ND, GW, RC, or MW) was also present at all times to manage any problems and answer questions. A stand consisting of three sets of two whiteboards was set up underneath a sunshade at each event, with a blackboard displaying information about the study to alert passers-by to our presence and purpose. Adults with children were also approached and provided with written and verbal information about the study and invited to take part. Once they approached the stand, adults and young people were invited to ask questions prior to participation. Written consent was completed by all adults including on behalf of the young person for whom they were responsible. We added an additional verbal consent with young people to ensure that they were informed about the study and agreed to participate. When interviewing, researchers made it clear to both adults and young people that consent could be withdrawn at any time, for any reason. We faced some difficulties recruiting 15- and 16-year-olds in particular, as they tended to attend the events without adults, which made it impossible to secure parental consent thus rendering them ineligible for the study.

Young people received a £5 Amazon voucher, a small bag of sweets, and a packet of football stickers as a token of appreciation for participation. We chose to give stickers from the Panini World Cup 2018 series which has no gambling sponsors so that we could ensure that they did not include gambling logos.

### Data collection

Prior to data collection, all researchers undertook 2-h training sessions (including piloting the questionnaires). This training was supplemented by a briefing at the beginning of each data collection site to ensure that all researchers were absolutely clear about their roles. Responses were checked by ND and GW for quality and consistency during lulls in activity and after each data collection period.

After consenting to the study, young people and adults were asked questions about their socio-demographic characteristics, including their age, gender, and ethnicity. Adults were also asked to provide their postcode so that we could assess the diversity of the sample using IMD [26] criteria. The Index of Multiple Deprivation [26] (IMD) was used as a proxy measure for socio-economic background calculated from postcode data supplied by the participating adult. IMD is an area level measure that synthesises data based on income, employment, health deprivation and disability, education, housing, and crime into relative deprivation levels that range from 1, representing the most deprived 10% of England, to 10, representing the least deprived 10%.

Adults were also asked about their recent participation in gambling as an indicator of gambling behaviour (ethical guidelines prevented this question from being asked to those under 16 years old). To get an indication of young people’s perception of their own proximity and exposure to gambling activity we asked ‘how many adults that you know, gamble on sport?’ All participants were then asked how many gambling brands they could name. Young people and adults were then separated to participate in the implicit recall activity.

There are 20 clubs in the EPL. During the 2017–18 season, nine teams’ shirts were sponsored by gambling brands. Four gambling and four non-gambling shirt sponsored teams were chosen for the magnet board activity. This meant that when picking the non-gambling control brands (10 in total), we had to choose some additional team sponsors from the Championship. To balance our selection, we decided on one gambling and one non-gambling shirt sponsored team relegated from the Premier League in 2016 (Aston Villa and Norwich City), and two further non-gambling sponsored teams Wigan Athletic and Bristol, and a similar-sized gambling sponsored team, Leeds.

Each participant was presented with a whiteboard which showed eight EPL team logos (2017–18 season). These were placed in a random, vertical order from top to bottom. Four of the teams had gambling-sponsored team shirts (Crystal Palace, Everton, Stoke City, West Ham), and four had non-gambling shirt sponsors (Arsenal, Chelsea, Leicester City, Manchester United).

Randomly ordered brand logos were placed at the bottom of the board. These 23 logos included the eight matching team shirt sponsors (ManBetX, SportPesa, BetWay, Bet365, Emirates, Yokohama Tyres, King Power, and Chevrolet respectively) along with 15 other gambling (Fun88, Unibet, 32 Red, OPE, Ladbrokes) and non-gambling brands (AIA, Inter Sport, American Express, Standard Chartered, Lancer Scott, Etihad, Palm, FXPro, Aviva, and Virgin Media) that sponsored other EPL and Championship teams.

A key difficulty when choosing the logos for the magnet board activity was the prevalence of secondary sponsorships—in particular the use of ‘betting partners’, stadium partners, shirt sleeves (new to 2017 season), and companies buying banners in grounds. Although Ladbrokes are not shirt sponsors, for example, they are official betting partners to numerous EPL clubs and listed by clubs as sponsors. As a result, Ladbrokes logos appear at many grounds and official events (for example, the backgrounds used at press conferences). We chose to include the Ladbrokes logo in order to investigate how this type of sponsor relationship was interpreted, if at all.

A ‘projective technique’ was used whereby participants were not asked directly to match teams and sponsors but simply asked to place as many or as few brand magnets as they wished anywhere on the whiteboard ([3], pg. 2). Each participant was asked why they had positioned the magnets as they had, and this information was recorded. Participants were then given four magnetic gold stars and invited to indicate their most preferred teams and their most preferred brands by placing a star next to each one. Each board was given a unique identifier, and the boards were photographed for later analysis.

Following the magnet board activity, each participant was asked a combination of discrete and open-ended questions including:

- Now that you have completed the magnet activity, do you remember seeing any advertisements or brand logos for betting during sports matches?
- What do you think about the advertising of betting during sports matches?
- Do you think betting has become a normal part of sport?
- Would you like to see any changes to the way betting is advertised during sporting events?

These questions were asked after the magnet board activity in order to reduce any impact that they might have otherwise had on that exercise. Participants were then asked where they had seen advertisements for betting companies, first unprompted, and then with the aid of a picture board which showed 12 different options including a television screen, computer screen, betting shop window, and billboard.

### Approach to analysis

Gridlines were drawn onto the whiteboards prior to data collection, to help researchers objectively assess the distance between the placed magnets and team logos. In order to produce comparable data, the layout, spacing, and analysis of proximity in relation to boxes followed the specifications from the Australian study [32]. All data were checked and cleaned before being coded. The main adjustments were small changes to the wording of qualitative data.

Quantitative data was analysed using IBM Statistical Program for Social Sciences (SPSS) 22.0. Chi-square tests of association were used to determine whether different groups of young people (based on age or gender) differed significantly, or not, in terms of their abilities to implicitly recall relationships and in their answers to survey questions. In order to assess the relationship between watching sport on television and exposure to gambling advertising, recall of gambling brands, and attitudes towards gambling during sporting broadcasts, young people and adults were asked how often they watched various sports on television, choosing from options which ranged from ‘never’ to ‘all of the time/as often as I can’. On the basis of this data, a category of football fan who watched football on television ‘all of the time/as often as I can’ was identified and referred to as ‘Super Fan’.

Qualitative responses were transferred to data management software NVivo and thematically analysed. Data interpretation was led by ND, with all coding checked by a second researcher after several re-readings. Regular meetings took place between all authors in order to discuss emergent themes. In this paper, qualitative responses are used primarily to complement quantitative data.

## Results

### Sample characteristics

A total of 99 young people and 71 adults took part in the study. Most of the young people (*n* = 71, 72%) were aged between 8 and 11 years (mean = 10.42, sd = 2.218), with most adults (*n* = 28) aged 36–45 (mean = 43.93, sd = 9.625). Two thirds of the young people who took part were boys (*n* = 66, 67%), and two thirds of the adults who participated were women (*n* = 47, 66%). The majority of the adults (*n* = 63, 89%) were parents of the participating young people, the others being grandparents or other carers.

According to their postcodes, about a third of young people (*n* = 31, 31%) and adults (*n* = 23, 32%) were from the top 20% of most deprived areas according to the IMD. Under half of young people (*n* = 44, 44%) and 39% of adults (*n* = 28) identified as black or minority ethnic (BME).

The majority of young people regularly played football (*n* = 79, 80%), with just over half (*n* = 55, 55%) playing multiple times in a week, or as part of a team. A majority of adults (*n* = 44, 62%) and three quarters of young people (*n* = 74, 75%) stated that they watched football more than any other sport, with over a quarter of adults (*n* = 20, 28%) and one third of young people (*n* = 33, 33%) saying that they watched football ‘all of the time/as often as I can’. The majority of young people (*n* = 82, 83%) and two thirds of adults (*n* = 47, 66%) said that they supported a football team.

Seven adults (10%) said that they had bet money on sports within the last week. Almost three quarters of adults (*n* = 52, 73%) and over half of young people (*n* = 54, 55%) stated that they knew adults who gambled on sports (Table 1).

**Table 1:** Sample characteristics

Characteristic	*n* (%)
Young people	
Age	
8–11 years	71 (72)
12–16 years	28 (28)
Gender	
Male	66 (67)
Female	33 (33)
IMD top 20% most deprived	31 (31)
Ethnicity	
BME	44 (44)
White	54 (55)
Regularly play football	79 (80)
Watch football more than other sports	74 (75)
Support a football team	82 (83)
Know adults who gamble	54 (55)
Adults	
Age	
18–25	0
26–35	15 (21)
36–45	28 (39)
46–55	17 (24)
56–65	10 (14)
65+	1 (1)
Gender	
Male	24 (34)
Female	47 (66)
IMD top 20% most deprived	23 (32)
Ethnicity	
BME	28 (39)
White	41 (58)
Regularly play football	10 (14)
Watch football more than other sports	44 (62)
Support a football team	47 (66)
Bet money in past week	7 (10)
Know adults who gamble	52 (73)

### Sports betting brand recall

Table 2 presents data on betting brand recall. Just under half of young people (*n* = 46, 46%) and more than two thirds of adults (*n* = 49, 71%) were able, unprompted, to name at least one gambling brand. Fourteen young people (*n* = 14, 14%) and a third of adults (*n* = 25, 33%) were able to name three or more gambling brands unprompted (child range 0 to 5, mean 0.96; adult range 0-6, mean 1.92).

**Table 2:** Betting brand recall

	Adults	All young people	Young people: ‘Super Fan’	Young people: female	Young people: male
Named no bet brands	20	53	10	25	28
Named one or more bet brands	49	46	23	8	38
Totals *n*	69	99	33	33	66
Significance			0.01	0.02	0.02
Top five brands reported		Brand named		*n* (%)	
Young people		Bet365		30 (30)	
		Betway		18 (18)	
		Ladbrokes		16 (16)	
		William Hill		13 (13)	
		Coral		4 (4)	
Adults		Ladbrokes		25 (35)	
		William Hill		24 (34)	
		Paddy Power		21 (30)	
		Bet365		20 (28)	
		Betfred		14 (20)	

Boys had a significantly higher recall of gambling brands compared to girls (*p* 0.02). Young people who were categorised as ‘Super Fans’ were also significantly more likely to be able to name one or more brands compared to other young people (*p* = 0.01). The brand most commonly recalled by young people was *Bet365* (*n* = 30), followed by *Betway* (*n* = 18). The top brands named by adults were Ladbrokes (*n* = 25) and William Hill (*n* = 24).

### Implicit recall of sponsorship

Almost two thirds of young people (*n* = 63, 63%) correctly placed one or more shirt sponsor magnets next to the corresponding football team. Just under a third (*n* = 30, 30%) correctly placed three or more shirt sponsors magnets next to the corresponding football team. Young people categorised as ‘Super Fans’ were significantly more likely to place a sponsor next to the correct team compared to other young people. More than half of ‘Super Fans’ (*n* = 17, 52%) were able to correctly place three or more shirt sponsor magnets next to the corresponding football team (Table 3). Boys correctly placed an average of 2.11 shirt magnets next to the corresponding football teams while girls averaged 0.73 matches. Just under two thirds of adults (*n* = 44, 62%) correctly placed one or more shirt sponsor magnets next to the corresponding football team. Women correctly placed fewer pairs on average (0.89) than men (3.17).

**Table 3:** Implicit recall of sponsorship

		Adults *n* (%)	Young people *n* (%)	Young people: ‘Super Fans’ *n* (%)
Number of teams matched to sponsors within 4 squares	Matched 0	27 (38)	36 (36)	7 (21)
	Matched 1	14 (20)	18 (18)	5 (15)
	Matched 2	11 (15)	15 (15)	4 (12)
	Matched 3 or more	19 (27)	30 (30)	17 (52)
Means		1.66	1.77	2.79
Total *n*=		71	99	33
Significance				0.012

Both young people and adults most frequently placed the correct sponsors next to the three teams at the top of the EPL: Arsenal (adults *n* = 39, young people *n* = 49), Manchester United (adults *n* = 23, young people *n* = 42), and Chelsea (adults *n* = 18, young people *n* = 31). One in five young people (*n* = 20, 20%) correctly placed one or more gambling logos next to the corresponding team (range 0–4, mean 0.32). Among adults, this was 28% (*n* = 20, range 0–3, mean 0.37).

### Rationale for magnet placement

Participants were asked to explain how they chose to place their magnets, and their responses were coded into eight themes. The top two themes for young people were that they identified the brand specifically as shirt sponsor (*n* = 27, 27%) or that they identified the brand as a team sponsor (*n* = 24, 24%). Shirt and team sponsorship were also the most common explanations for magnet placement given by Super Fans (*n* = 12, 36% for each of these two themes). Young people thought explicitly about the association between brands and teams during the task, as one 8-year-old boy explained:

*I watch football a lot and I see what the sponsor on the shirt is and I kind of remembered it and I think of it and put it next to the team.*

One fifth (*n* = 20, 20%) of young people used ‘random’ positioning. This rationale was given far more often by the younger age group and by girls than it was by the older age groups, boys, and ‘Super Fans’. Adults most frequently placed brand magnets by identifying them as team sponsors (37%, *n* = 26) or shirt sponsors (*n* = 11, 15%). Random positioning was used by one fifth of adults (*n* = 14, 20%), the same proportion as young people (Table 4).

**Table 4:** Reasons given for placements of brand magnets

	Adult *n* = 71	Young person *n* = 99	Male young person *n* = 66	Female young person *n* = 33	Super fan young person *n* = 33	Young person aged 8–11 *n* = 71	Young person aged 12–16 *n* = 28
Identified brand specifically as shirt sponsor	11	27	22	5	12	18	9
Identified brand as team sponsor	26	24	21	3	12	18	6
Associated brand with team but were unsure of the brands relationship to team	8	15	11	4	5	11	4
Associated brand with football in general	8	19	14	5	5	18	1
Similar colours	4	12	6	6	1	9	3
Remembered seeing brand at stadium	2	11	8	3	6	10	1
Recognised and were familiar with the brand	6	13	9	4	5	12	1
Randomly placed the magnet	14	20	13	7	4	14	6
Other, including unable to provide an explanation	14	17	10	7	5	10	7
Totals	71	99	66	33	33	71	28

### Most preferred (starred) brands

A quarter of young people (*n* = 25, 25%) placed a star next to a gambling brand. The gambling brands most starred by young people were *Bet365* (*n* = 10) and *Betway* (*n* = 10). Reasons given by young people for their choice of brand to star included the familiarity of the brand. For example, a 15-year-old girl ‘recognised’ *Bet365* and chose to star it. A 13-year-old boy also chose *Bet365* saying that it was the *‘most common one he had heard of’*.

Among adults, 20% starred a gambling brand (*n* = 14). The most starred gambling brands were *Bet365* (*n* = 5) and *Ladbrokes* (*n* = 5). Reasons given by adults for the choice of brand to star included *‘I starred Ladbrokes because I thought it was Everton’s sponsor’* (33-year-old female Everton fan), *‘starred the company I bet with’* (33-year-old male), and *‘I chose the two brands I know best to star: Intersport and Ladbrokes.’* (41-year-old woman).

### Recall of gambling advertising in different media environments

Without prompting, young people most frequently recalled seeing gambling advertisements on television (*n* = 45), followed by betting venues (*n* = 15), and on technology/screens (*n* = 14). Young people are not allowed into betting venues in the UK but explained they had seen adverts in ‘shop windows’. After viewing the picture board of potential gambling advertising locations, the most common places young people recalled seeing gambling advertising were overwhelmingly on television (*n* = 78), followed by technology/screens (*n* = 49) and sports teams (*n* = 43).

Adults had unprompted recall of seeing gambling advertising on television (*n* = 43), technology/screens (*n* = 16), and sports teams (*n* = 16). After seeing the location picture board, television was still the most frequently recalled places (*n* = 56), followed by technology/screens (*n* = 37), with sports stadiums (*n* = 34) and betting venues (*n* = 34) joint third (Table 5).

**Table 5:** Recall of locations where gambling advertising was seen by adults and young people

Where were gambling ads seen?	Adults = 71	Young people = 99	Young people male = 66	Young people female = 33	Young people ‘Super Fans’ = 33	Un-prompted adult	Un-prompted young people
Television	56	78	55	23	27	43	45
Bus stops	15	28	20	8	13	7	6
Sport teams	30	43	30	13	16	16	13
Train	18	13	9	4	4	5	9
Bill boards	29	38	23	15	18	15	10
Sports stadium	34	36	23	13	13	15	9
Public transport	25	21	13	8	9	12	8
Radio	27	22	16	6	8	6	5
Newspapers	28	29	21	8	12	8	9
Betting venue	34	29	20	9	11	15	15
Social media	32	35	20	15	15	12	7
Technology/screens	37	49	34	15	18	16	14

### Perceptions of the normalisation of gambling in sport

More than three quarters of young people (*n* = 74 out of 95 responses, 78%) considered betting to be a normal part of sport. Seventy-two percent of young people (*n* = 68, out of 95 responses) thought betting on sport was a normal thing for adults to do, with 13% (*n* = 12, out of 94 responses) thinking it was normal for young people. When young people were asked how many people under 16 years old they thought gambled on sports, just over half thought none (*n* = 49) and about half (48%) believed at least some participated in gambling to varying levels (‘one or two’ *n* = 22, ‘a few’ *n* = 19, ‘a lot’ *n* = 4). Almost three quarters of young people (*n* = 68, out of 93 responses, 73%) thought that 16 and 17 year olds were betting illegally on sports. When asked how many 16 and 17 year olds bet on sports, six answered ‘one or two’, 37 answered ‘a few’, and 25 thought ‘a lot’. In discussing the normality of sports betting, young people commonly mentioned informal betting among friends and betting as a way of supporting their team:

*It’s normal for under 18’s to bet with friends. I put £2 on Man U with other young people.* (13-year-old male)

*‘Because if they love football it (betting) is part of wanting their team to win.’* (8-year-old male)

The majority of adults (*n* = 59 out of 69 responses, 86%) thought that betting had become a normal part of sport. Almost three quarters thought that betting on sports was a normal thing for adults to do (*n* = 50, out of 70 responses, 71%), and 20% thought it was a normal thing for young people to do (*n* = 14, out of 70 responses). Although most adults thought that is was not normal for young people to bet on sports, three quarters of them (75%) thought that 16- and 17-year-olds gambled on sports to some extent (*n* = 53; ‘one or two’ *n* = 7, ‘a few’ *n* = 31, ‘a lot’ *n* = 15), and 45% thought this was also the case for under 16 year olds (*n* = 32; ‘one or two’ *n* = 6, ‘a few’ *n* = 20, ‘a lot’ *n* = 6). Many adults specifically noted how widespread and acceptable sports betting had become, particularly in relation to football:

*'You associate it with sports, especially football now.'* (48-year-old female)

'(Betting) *is much more acceptable. Betting shops need to be dark alien- unknown places, non-attractive, not advertising with famous people.'* (48-year-old male).

### Attitudes towards the advertising of betting during sport

Young peoples’ opinions about the advertising of betting during sport varied: 31% (*n* = 31) were negative, 27% (*n* = 23) were positive, 23% (*n* = 23) were neutral, and 18% (*n* = 18) were unsure. The reasons given by young people who were negative about betting advertising during sport included:

*'It makes people want to use their money and leaves them broke. It’s an addictive habit.'* (13-year-old male)

'*Some kids watch sport and it influences them to bet and they might want to do it.*' (13-year-old-female).

Young people who were positive about betting advertising during sport demonstrated a misperception of betting as a risk-free way to win money. Comments included: *‘you can get money for winning’* (11-year-old boy); *‘I like it. I want to win money’* (8-year-old boy); and *‘it’s good, you get loads of money’*. (8-year-old boy). They also made an association between betting and supporting a team: ‘sponsorship supports the club so is a good thing’ (11-year-old boy).

The majority of adults (51% *n* = 36) had negative opinions about betting advertising during sport. One 48-year-old female commented:

*It’s annoying. It doesn’t give a good message and shouldn’t be associated with the things my son loves.*

One fifth of adults (*n* = 15, 21%) were neutral, and almost one fifth (*n* = 13, 18%) were positive about betting advertising during matches including a 47-year-old female who said:

*It is fine, companies advertising- it is just business.*

### Attitudes towards harm reduction strategies

The majority of adults (*n* = 57, 80%) and young people (*n* = 56, 62%) believed that betting advertising during sports matches had an impact on young people’s betting behaviour. Television advertising was identified by both adults (*n* = 38) and young people (*n* = 30) as having the most impact, with adults naming ‘social media’ (*n* = 22) and young people ‘technology/screens’ (*n* = 24) second:

'*Most young people see advertisements on television, more often than other places.*' (9-year-old male)

'*More young people are on gadgets and on their phone, and adverts keep popping up, and they will think betting is good and spend money a lot then*' (13-year-old male)

Adults referred to the intrusiveness of gambling advertising on social media ‘There is no censoring- they pop up everywhere’ (44-year-old female). Others expressed concern about advertisements on television which they felt appealed to children:

B*ecause they capture the young people’s attention with funny dancing or someone famous, or funny music. It catches young people’s attention and they remember it.* (29-year-old female)

When asked which form of advertising, if any, should be banned, adults (*n* = 26) and young people (*n* = 18) predominantly identified television advertising. When asked what specific changes to advertising they would like to see, the top two options chosen were ‘remove sports betting advertising in social media’ (adults *n* = 41, young people *n* = 38) and ‘remove sports betting advertising during television matches shown before 9 pm’ (adults *n* = 39, young people *n* = 36).

## Discussion

The study is a first step in the process of addressing the lack of evidence about young people and gambling advertising in the UK using methods developed in Australia. Australian studies have demonstrated that over three quarters of young people can name one or more gambling brands ([3], pg. 4) compared to just under half of young people in the present study. However, this comparison should be interpreted cautiously as the Australian study deliberately targeted sports fans and participants, whereas this study examined a broader community sample of young people and adults. The more direct comparison might be with the ‘Super Fans’ of whom over two thirds could name one or more gambling brand. Similar to the Australian research, our study indicated that older children were able to name more brands than younger children. However, while Australian studies have demonstrated that there are few differences in gambling brand recall according to gender [3], in our study, boys had a significantly higher recall compared to girls, with boys as young as eight able to name three brands. These findings, along with a recent report by the Gambling Commission [27], which found that boys have a higher rate of problem gambling (2.0%) than girls (1.3%), suggest that a gendered approach to harm prevention may be warranted, including a closer examination of the relationship between gender, supporting/playing football, and gambling behaviour/attitudes ([28] in press).

As with findings for unprompted brand recall, young people who watched a lot of football on television (Super Fans) were significantly more likely to place a sponsor next to the correct team compared to other young people; indeed, over half were able to correctly place three or more shirt sponsors magnets next to the corresponding EPL team. As with unprompted recall of betting brands, boys and men made more correct matches between sponsors and teams than girls and women, again raising questions about the gendering of engagement with football and its impact on implicit awareness and behaviour.

Throughout the study, qualitative responses from young people showed that some of them conflated support for the brand with support for the team. The brands most ‘starred’ by young people were both companies with well-established associations with EPL football—*Bet365* owns and has sponsored Stoke City since 2012 and *Betway* has sponsored West Ham since 2015. Proximity to West Ham does not appear to account for this finding—Crystal Palace was the closest EPL stadium to the research sites and no one recalled their shirt sponsor (gambling brand ManBetX) at the start of the survey. Moreover, only three adults and seven children correctly matched this team with their shirt sponsor. However, in a recent study, *Bet365* was the most advertised brand in coverage of EPL football on the BBC, while *Betway* was the most advertised during coverage of the EPL on Sky television [11]. Clubs and leagues that wish to promote family-friendly products should consider following Luton Town’s example [29] and opting out of sponsorship deals with gambling companies, as has also happened in Australia [24] and Ireland [30].

Research has shown that normalisation occurs when young people are exposed to the marketing of adult products including tobacco, alcohol, and gambling and that this can affect both their intentions to consume and actual consumption of these products [2, 31, 32]. Our findings are very similar to those of Australian studies which demonstrate that the majority of young people and adults perceive that gambling is becoming a normal part of sport [33]. The similarity of these and other findings suggests that it may be useful for policymakers to use the principle of ‘logic based on parallel evidence’ [13], utilising the evidence from Australia to guide policy and public health prevention responses while they wait for research to be produced in the UK. It is difficult to predict how the perception of betting as a normal part of sport will affect the future consumption behaviour of young people. However, a growing body of parallel evidence from other jurisdictions suggests that it is likely to influence the uptake of betting. For example, one study in Australia found that young people who watch a lot of sport on television are more likely to say that they would like to try gambling when they grow up [34].

In our study, the vast majority of young people stated that they had seen gambling advertising on television. This mirrors a survey of 2881 11–16-year-olds in Great Britain by the Gambling Commission, which found that 80% of 11- to 16-year-olds had seen gambling advertisements on television and 39% had seen them more than once a week [35]. These findings raise questions as to the effectiveness of existing regulations, including the 9 pm watershed, and also the appropriateness of the exception which allows sports betting firms to advertise during live broadcasts of sports. While a ban on advertising during live sport has been widely suggested and supported in the UK [25], including by some bookmakers, our data suggests that this single measure may not be sufficient to significantly reduce young people’s exposure to gambling advertising, which they also reported seeing in a range of media, sporting, and land-based environments. A recent study of broadcasts of EPL football on the BBC and Sky found that the majority of the instances of advertising appeared on dynamic billboards surrounding the pitch [36].

Despite the fact that gambling advertising has been banned from media designed for young people since 2007, the Gambling Commission survey of 2881 11–16-year-olds found that 70% of young people say they have seen advertisements on social media and 66% on other websites [35]. Figures were lower among our participants, perhaps because our sample included younger children. Even so, half of young people recalled having seen gambling advertisements on screens and technology and over a third had seen them on social media. This suggests that changes to the regulation of gambling advertising during commercial breaks on television alone may not prevent the exposure of young people to gambling advertising. A recent study in Australia examining the efficacy of the new rules which banned gambling advertising during live sport broadcast on television before 8.30 pm found that young people continue to watch live sport on television after 8.30 and were exposed to gambling advertising on other channels, including social media [21]. The study highlighted the need for urgent and comprehensive regulatory responses to address the range of channels and mechanisms that the gambling industry may use to promote their products.

Our study suggests that gambling advertising on television and social media is a matter of concern for some young people and adults. Both groups felt that it had an impact on young people’s behaviour and described the intrusiveness of gambling advertising and its ability to capture attention using a range of techniques including by featuring celebrities and sports people, humour, and catchy music. The content of adverts in the UK and their impact on young people in particular was raised as a concern and requires further investigation, as has already taken place in Australia [2].

## Limitations

The lower numbers of 14 to 16-year-olds in this study reflects the difficulty of recruiting young people for this age group, as they tended to attend events with their peers rather than with adults. Data was collected during the FIFA World Cup when both football and gambling advertising were particularly prevalent. Young people may have been exposed to more advertising as a result. Further studies at different times in the football season may help us to understand whether or not the volume of advertising that accompanies any important event was a factor in our findings, and if so, how it affected the responses of young people and adults. We anticipated that the magnet board activity could potentially trigger recollection of gambling brands and therefore asked participants to recall as many brands as they could before engaging in the task. However, there is also a chance that the magnet board activity affected the answers given to subsequent questions. If the study was repeated, counterbalancing could be considered for the task/question order.

## Conclusions

Young people in the UK are exposed to gambling advertising via a range of media and through sponsorship and promotion as well as advertisements. While a partial ban on televised advertising during live sport has been widely suggested and supported in the UK [25], our data suggests that this single measure may not be sufficient to significantly reduce young people’s exposure to gambling advertising. Policymakers should consider comprehensive approaches, such as those applied in tobacco control, which take into consideration all forms of advertising, including promotion and sponsorship. Policymakers should also consider how recent changes in Australia have been more or less successful in reducing the exposure of young people to gambling advertising.
